# Analysis of Melanoma Secretome for Factors That Directly Disrupt the Barrier Integrity of Brain Endothelial Cells

**DOI:** 10.3390/ijms21218193

**Published:** 2020-11-01

**Authors:** Akshata Anchan, Olivia Martin, James J. W. Hucklesby, Graeme Finlay, Rebecca H. Johnson, Laverne D. Robilliard, Simon J. O’Carroll, Catherine E. Angel, E Scott Graham

**Affiliations:** 1 Department of Molecular Medicine and Pathology, Faculty of Medical and Health Sciences, University of Auckland, Auckland 1023, New Zealand; a.anchan@auckland.ac.nz (A.A.); olivia.martin@auckland.ac.nz (O.M.); james.hucklesby@auckland.ac.nz (J.J.W.H.); g.finlay@auckland.ac.nz (G.F.); l.robilliard@auckland.ac.nz (L.D.R.); 2 Centre for Brain Research, Faculty of Medical and Health Sciences, University of Auckland, Auckland 1023, New Zealand; rebecca.johnson@auckland.ac.nz (R.H.J.); s.ocarroll@auckland.ac.nz (S.J.O.); 3 School of Biological Sciences, Faculty of Science, University of Auckland, Auckland 1010, New Zealand; c.angel@auckland.ac.nz; 4 Auckland Cancer Society Research Centre, School of Medical Sciences, Faculty of Medical and Health Sciences, University of Auckland, Auckland 1023, New Zealand; 5 Department of Pharmacology, School of Medical Sciences, Faculty of Medical and Health Sciences, University of Auckland, Auckland 1023, New Zealand; 6 Department of Anatomy and Medical Imaging, School of Medical Sciences, Faculty of Medical and Health Sciences, University of Auckland, Auckland 1023, New Zealand

**Keywords:** melanoma, brain endothelial cells, ECIS technology, cytokine profiling, metastasis, TGFβ, ANGPTL-4

## Abstract

We have recently demonstrated that invasive melanoma cells are capable of disrupting the brain endothelial barrier integrity. This was shown using ECIS biosensor technology, which revealed rapid disruption via the paracellular junctions. In this paper, we demonstrate that melanoma cells secrete factors (e.g., cytokines) that weaken the endothelial barrier integrity. Through proteome profiling, we attempt to identify the barrier-disrupting cytokines. Melanoma conditioned media were collected from three New Zealand melanoma lines. ECIS technology was used to assess if the conditioned media disrupted the endothelial barrier independent of the melanoma cells. The melanoma cell secretome was assessed using cytometric bead array (CBA), Luminex immunoassay and multiplex Proteome Profilers, to detect the expression of secretory proteins, which may facilitate metastasis. Finally, ECIS technology was used to assess the direct effects of secreted proteins identified as candidates from the proteome screens. We show that melanoma-conditioned media significantly disrupted the brain endothelial barrier, however, to a much lesser extent than the cells from which they were collected. Cytokine and proteome profiling of the conditioned media showed evidence of high concentrations of approximately 15 secreted proteins (including osteopontin, IL-8, GDF-15, MIF and VEGF). These 15 secreted proteins were expressed variably across the melanoma lines. Surprisingly, the addition of these individually to the brain endothelial cells did not substantially affect the barrier integrity. ANGPTL-4 and TGFβ were also produced by the melanoma cells. Whilst TGFβ-1 had a pronounced effect on the barrier integrity, surprisingly ANGPTL-4 did not. However, its C-terminal fragment did and within a very similar period to the conditioned media, albeit not to the same extent. Herein we show that melanoma cells produce a wide-range of soluble factors at high concentrations, which most likely favour support or survival of the cancer cells. Most of these, except for TGFβ-1 and the C-terminal fragment of ANGPTL-4, did not have an impact on the integrity of the brain endothelial cells.

## 1. Introduction

We have recently demonstrated that a panel of patient-derived melanoma lines disrupt the barrier integrity of human brain endothelial cells in a manner that rapidly targets the paracellular junctional space [1]. This was demonstrated using ECIS biosensor technology in combination with live-cell imaging. This indicated that the melanoma cells were able to disrupt the junctional proteins, which maintain endothelial barrier integrity. The rate of this disruption was fast, which implied direct cell-cell mediated effects or the local production of secreted factors (e.g., cytokines) by the melanoma cells that then weaken the endothelial barrier integrity [1].

Cancer metastasis is enabled by secretory cytokines, chemokines and growth factors. It is characteristic of cancer to alter and abuse natural physiological pathways for neoplastic progression. These soluble factors are responsible for the cross-talk between melanoma, macrophages, and endothelial cells in the primary tumour site. This suggests their importance in mediating melanoma–endothelial interactions at the point of extravasation at the secondary tumour site. The role of secretory molecules is well defined in leukocyte extravasation at the endothelium. During leukocyte extravasation, the cytokines TNFα and interferon γ (IFNγ) activate re-arrangement of tight-junction proteins and increase the expression of chemokines such as MCP-1, IL-8, MIP-1α, and IL-6 amongst other CCLs and CXCLs to facilitate extravasation [2,3]. TNFα and IL-1β increase the expression of endothelial adhesion molecules including selectins, ICAM-1 and VCAM-1 [4,5] that mediate leukocyte extravasation. Interleukin-1 beta (IL-1β) also facilitates the release of VEGF by neighbouring cells [4] to evoke re-organisation of the endothelium.

Secreted proteins make a large cohort of such contributing factors that are responsible for cancer proliferation, survival and metastasis. Collectively, these factors are referred to as metastatic mediators and prime examples include VEGF, TGFβ, chemokines (e.g., IL-8, MCP-1), and various other cytokines (e.g., IL-1β, TNFα, and angiopoietins) produced by the respective cancer cells and tumour-associated cells. Vascular endothelial growth factor (VEGF) is a potent regulator of angiogenesis [6] that in cancer, promotes leaky vascularisation of the tumour microenvironment [7,8].

Several isoforms of VEGF are known and their expression can alter blood vessel patterning and promote tumour growth and survival in mice [9,10]. Furthermore, increased VEGF serum levels correlate with clinical tumour progression and survival of malignant melanoma cells [11]. Both cancer cells and tumour-associated cells secrete VEGF and TGFβ-1 to facilitate melanoma migration. TGFβ-1 can regulate extracellular matrix components (ECM) by inducing matrix metalloproteinases [12,13]. MMPs facilitate melanoma invasion and activate fibroblasts in the tumour microenvironment. Activated fibroblasts, in turn, support tumour proliferation [14,15] and also secrete MMPs for ECM remodelling to provide a scaffold for melanoma migration [14]. Other roles of TGFβ-1 include initiating epithelial-to-mesenchymal transition (EMT) [16,17] and signalling endothelial-to-mesenchymal transition (EndMT), which in turn increases the cancer-associated fibroblast tumour load [18,19]. Moreover, TGFβ-1, amongst other serum factors, can also influence the release of IL-8 in melanoma via the transcription factor NFκB [20]. IL-8 is also associated with multiple stages of melanoma metastasis and has previously been discussed for its ability to induce melanoma metastasis [21] and ECM remodelling through the MMP pathway [22].

The aim of this research was to determine whether melanoma cells secrete metastatic mediators that directly influence the integrity of the brain endothelium, in the context of weakening the barrier or facilitating invasion. We hypothesised that the melanoma cells would secrete a range of factors known to influence the vascular endothelium.

## 2. Results

### 2.1. Effect of Melanoma Conditioned Media on Brain Endothelium

Conditioned media were collected from melanoma cells (NZM7, NZM48, NZM74) on alternate days, for a duration of 7 days. This was done to specifically assess whether the cells basally released soluble factors (e.g., cytokines, chemokines) that affect the endothelial barrier strength. ECIS technology was used to measure any change in brain endothelial barrier integrity upon addition of the melanoma conditioned media.

**Figure 1:**
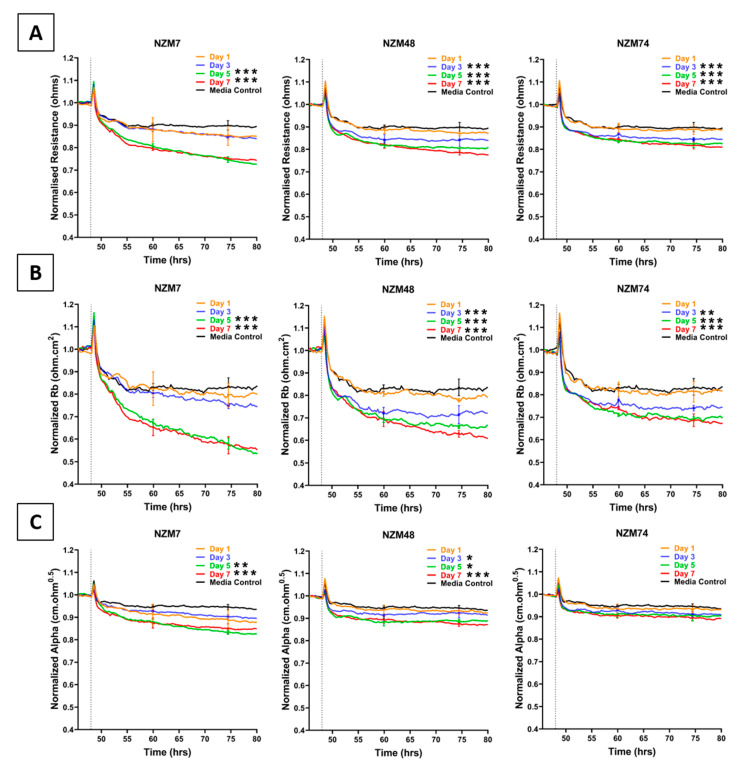
Effect of melanoma conditioned media on brain endothelial barrier resistance measured using ECIS technology. (**A**): shows unmodelled resistance (at 4000 Hz), (**B**): shows modelled Rb (paracellular resistance), (**C**): shows modelled Alpha (basolateral resistance) of hCMVECs over time after addition of conditioned media from three different NZM lines. Conditioned media was added at 48 h (dotted line). αMEM is media only control. Data shown as mean ± SD (*n* = 3 wells) from a single experiment. Data are representative of at least three independent experiments. End-point values from at least three independent experiments were compared relative to their media control using two-way ANOVA with Tukey’s range test as detailed in Methods (* *p* < 0.05, ** *p* < 0.01, *** *p* < 0.001).

Conditioned media collected from the three melanoma lines decreased endothelial barrier resistance compared to control media (Figure 1), but to a lesser extent than the direct addition of the melanoma cells [1]. This decrease was evident in the overall barrier resistance (Figure 1A). ECIS data can also be modelled into Rb and Alpha [23], which indicate the resistance relating to the paracellular barrier and the basolateral adhesion of the endothelial cells, respectively. It is clear from Figure 1B,C that the conditioned media has a much greater effect on the paracellular barrier (Rb) and a much smaller effect on Alpha. Notably, conditioned media collected from later days induced a more significant decrease in barrier resistance than conditioned media collected from earlier days. Media collected for NZM7 mediated the greatest reduction in barrier resistance.

These results were intriguing as they supported the prevailing theory that melanoma cells could facilitate their migration and invasion using secreted factors to weaken endothelial barriers. To investigate this, the melanoma conditioned media was examined for the expression of a range of secreted molecules.

### 2.2. Characterisation of Melanoma Conditioned Media

Melanoma conditioned media was assessed for the expression of selected secretory molecules known to aid cancer progression or vascular modulation using cytometric bead array (CBA) and Luminex immunoassay (see Table 1). CBA is a highly sensitive method for measuring secreted protein concentrations, and hence it was used to detect these key molecules of interest.

**Figure 2:**
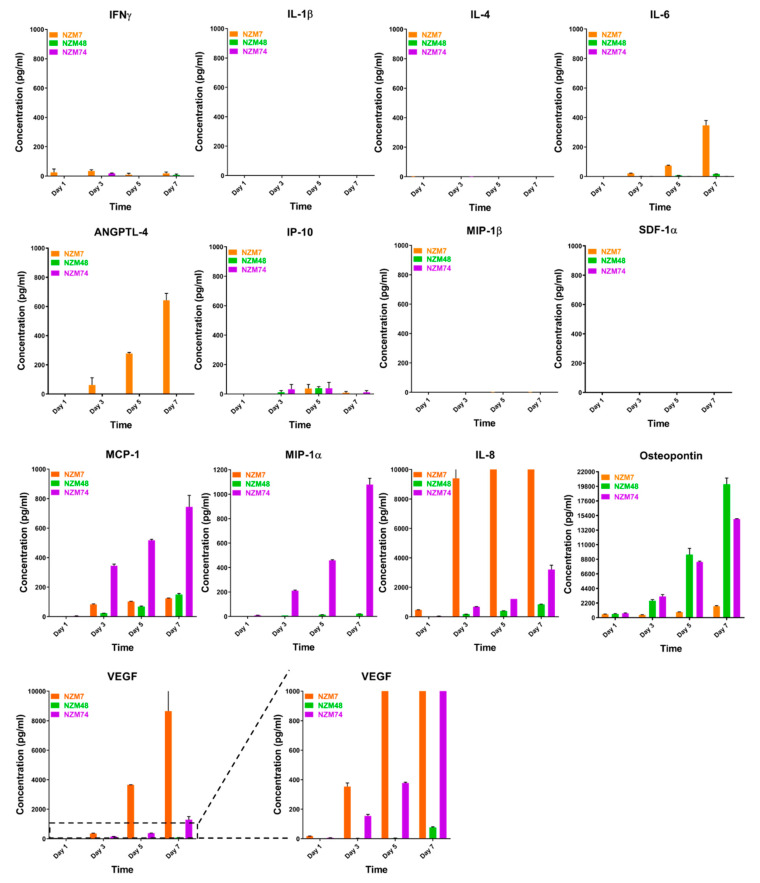
Quantitative expression of secreted factors in melanoma conditioned media. Concentration of 13 selected secreted factors of interest in melanoma conditioned media over time was measured by CBA or Luminex. Three NZM lines were tested. Conditioned media was collected from Day 1 (day of cell passage) on alternate days until Day 7 (~90% confluent). Note that for IL-8 and VEGF, the Y-axes are cut-off at max reliable detection range. Data are from one experiment showing the mean ± SEM (*n* = 3), representative of at least two independent experiments.

Of the 13 molecules assessed, IL-1β, IL-4, MIP-1β, and SDF-1 were not detected in the melanoma conditioned media (Figure 2). IFN-γ, IL-6, IL-8, MCP-1, MIP-1α, IP-10, osteopontin, ANGPTL-4, and VEGF were detected in the conditioned media but mostly at relatively low levels. IL-8, osteopontin and VEGF concentrations were considerably higher than the others and were produced by each of the melanoma lines. Concentrations of all detected cytokines increased with days in culture. These results correspond with the ECIS responses, which showed that the conditioned media collected on day 7 had a greater effect on weakening the endothelial barrier strength.

ANGPTL-4 was detected in conditioned media collected from NZM7 cells only. Notably this was at substantially lower levels than IL-8, osteopontin and VEGF. This was surprising as ANGPTL-4 is implicated in regulating vascular permeability with both pro- and anti-angiogenic roles (reviewed in [45]). ANGPTL-4 requires upregulation by TGFβ in many cancers [46,47,48] including melanoma metastasis to the brain [49]. Therefore, TGFβ-1 expression was also assessed in the melanoma conditioned media. However, we could not reliably detect the TGFβ-1 standard provided in the CBA kit, suggesting a failure of the kit. These findings have been communicated to the kit manufacturer. It was fully expected that TGFβ-1 would be expressed by the melanoma lines. Therefore, NanoString nCounter gene expression assay (detailed in the Methods section) was used to detect TGFβ-1 transcripts in cultured melanoma cells instead. TGFβ-1 is one of three isoforms of human TGFβ, which typically has the highest RNA expression in melanoma as collated by The Cancer Genome Atlas (TCGA [50] available from Human Protein Atlas, http://www.proteinatlas.org).

**Figure 3:**
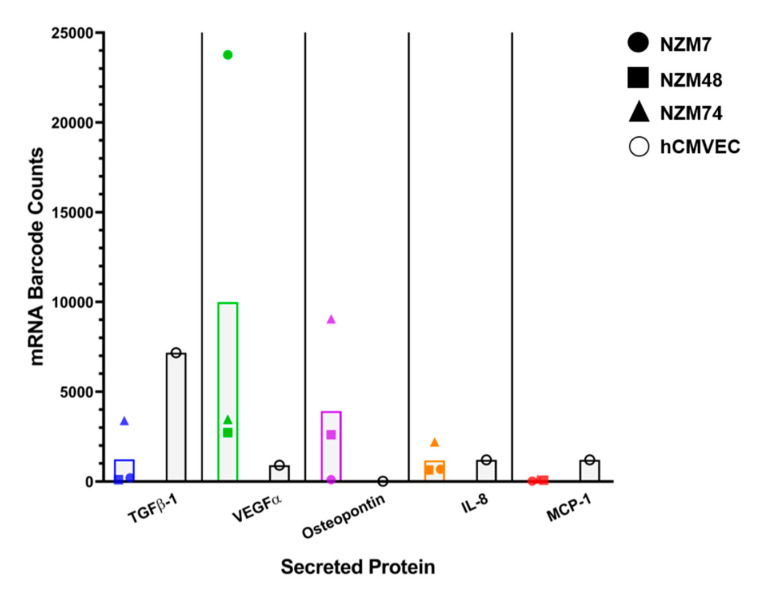
NanoString analysis of the expression of TGFβ-1 mRNA transcripts in melanoma and brain endothelial cells. Coloured points show transcript expression of three different NZM lines per secreted protein. Black points show expression in the brain endothelial cells for the same secreted proteins.

The NanoString assay uses molecular barcodes and images single molecules to detect and count hundreds of unique transcripts in a single sample. Figure 3 shows the mRNA barcode counts for TGFβ-1 as well as other highly expressed molecules such as IL-8 and VEGF as collected from confluent cultures of the three melanoma lines (NZM7, NZM48, NZM74 and the brain endothelial cells, hCMVECs–black bars). VEGF, IL-8 and osteopontin were added to our Nanostring dataset as a comparison for TGFβ. These proteins were selected as they were highly expressed on CBA and we expected TGFβ to match these levels. MCP-1, which was expressed but at relatively lower concentrations, was also added. There was evidence of TGFβ-1 transcripts in the melanoma cells; where these counts were lower than those detected for VEGF and osteopontin. The transcript levels for osteopontin were relatively low for the melanoma line NZM7 (mRNA barcode count: 97.18), which was interesting as osteopontin was detected in the conditioned media, however, at lower concentrations than NZM48 and NZM74. Similarly, IL-8, which was detected at relatively high concentrations in the conditioned media, also had relatively low mRNA counts in all melanoma lines, demonstrating that transcript and protein expression levels do not necessarily correlate. Intriguingly, brain endothelial cells also showed evidence of TGFβ-1 gene expression (black bars), and less so for VEGF, IL-8 and MCP-1. These results suggested that both melanoma and brain endothelial cells may produce TGFβ-1.

### 2.3. Common Mediators in Oncology and Inflammation

The secreted proteins assessed only make up a small proportion of secretory molecules, which may mediate cancer progression. The lack of expression of some of these molecules ruled these candidates out and suggested that we needed a broader approach. Therefore, expression of a wide range of inflammatory and oncologic secretory molecules was examined in a semi-quantitative manner using the Proteome Profiler XL Cytokine and Oncology screening kits (R&D systems). Note that all proteins assessed via CBA were also assessed via the Proteome Profilers to cross-check our responses and we are reasonably confident that the trend of expression is matched between the two assays, particularly for highly expressed molecules such as IL-8, VEGF and osteopontin, as well as ANGPTL-4. The Proteome Profiler screening assay was conducted using conditioned media collected after Day 7 of culture as it had the greatest amount of brain endothelial barrier disrupting activity.

**Figure 4:**
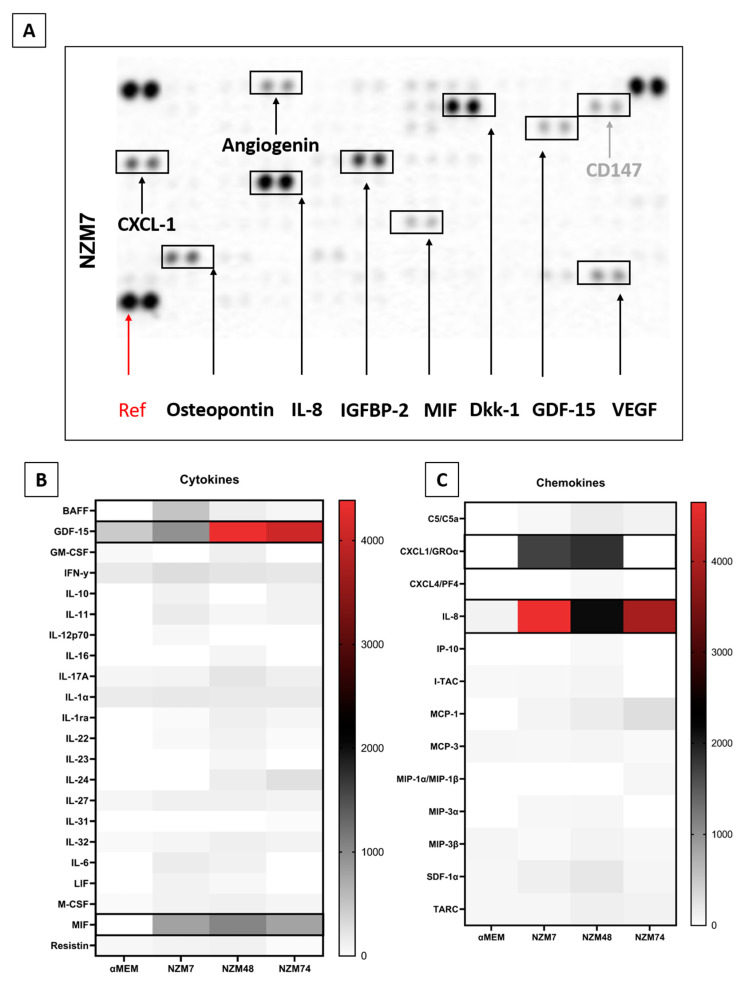

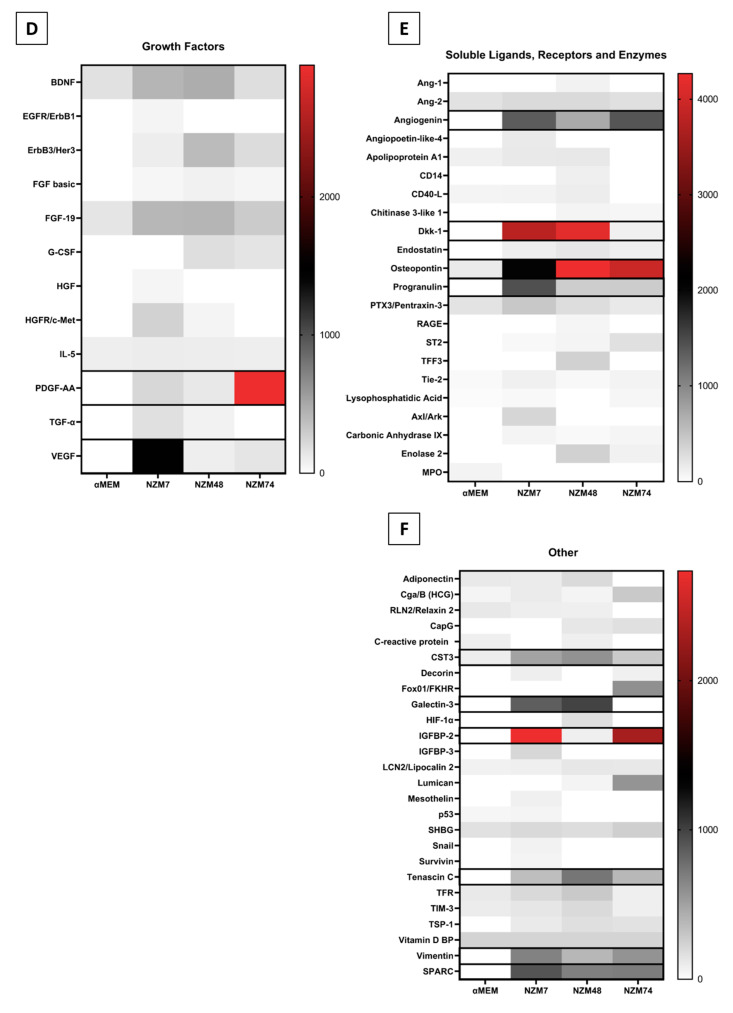
Proteome Profiler analysis of soluble factors present in the melanoma conditioned media. (**A**): shows an example of the spot intensity from the conditioned media on the XL Cytokine array. Heat maps are shown for (**B**): cytokines, (**C**): chemokines, (**D**): growth factors, (**E**): soluble ligands and receptors, and (**F**): other miscellaneous factors. The heat maps are colour-coded where the red is the highest expression and white is the lowest expression. Those present at consistently high levels across melanoma cultures are highlighted (black boxes).

An example of this assay is shown in Figure 4A, where the spot intensity correlates with cytokine abundance. This is a highly useful approach to screen samples for a broad range of factors simultaneously. Of the 142 different molecules assessed (across the XL Cytokine array and Oncology array), 95 were detected and are summarised in Figure 4B–F heat maps. They are divided into categories of (b) cytokines; (c) chemokines; (d) growth factors; (e) soluble ligands, enzymes and receptors; and (f) others (not classified in a previous group). The reasoning herein was to add more secreted proteins that may facilitate cancer metastasis, to our pre-existing list from Figure 2 and Figure 3.

We next focused on the secreted factors produced at moderately high levels in two or more of the melanoma cultures. These included GDF-15, MIF, osteopontin, angiogenin, CXCL-1, DKK-1, IGFBP-2, cystatin C, galectin-3, progranulin, SPARC, IL-8, tenascin C, and VEGF. In addition, we added ANGPTL-4 and TGFβ-1 from the previous analysis, and the cleaved C-terminal fragment of ANGPTL-4, which in the literature is shown to mediate the TJ disruption observed by ANGPTL-4 [24]. We also added SPARC like 1, which has a similar structure and overlapping functions to SPARC [51]. Many of these factors have been suggested to have roles in various stages of melanoma progression, and some have conflicting roles, which may indicate their differential expression between melanoma lines. Their functions and associations are described and summarised in Table 2.

### 2.4. Effect of Selected Secretory Molecules on the Brain Endothelium

**Figure 5:**
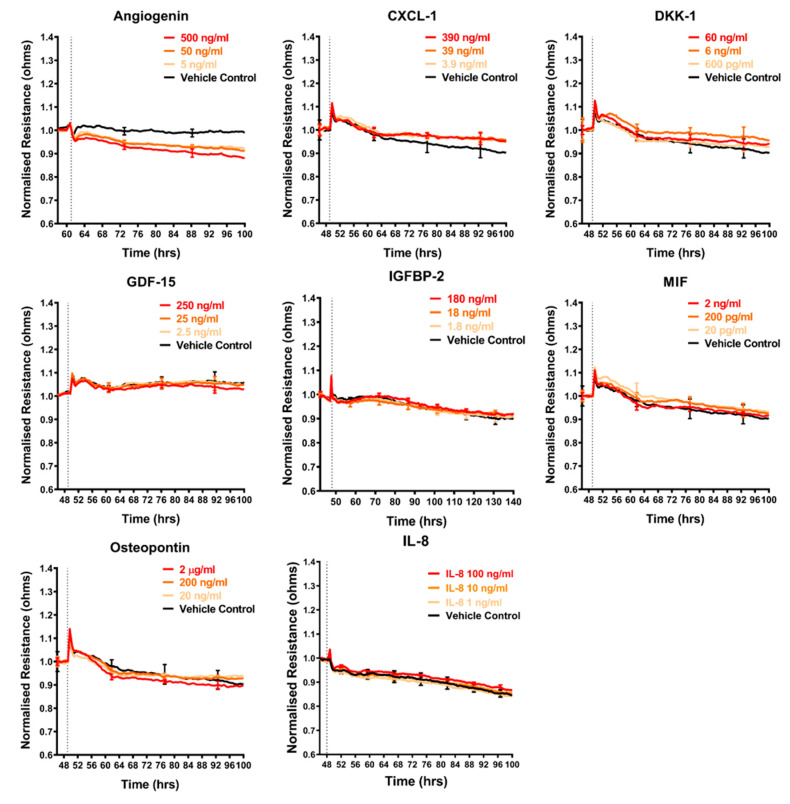
Effect of secreted protein on brain endothelial barrier resistance. Normalised unmodelled resistance (at 4000 Hz) of hCMVECs over time after the addition of eight highly expressed secreted proteins. Recombinant proteins were added (dotted line) at top concentrations as recommended by the supplier and the literature in a dilution series of 1:10. Data show the mean ± SD (*n* = 3 wells) from one experiment, which is representative of two to three independent experiments.

**Figure 6:**
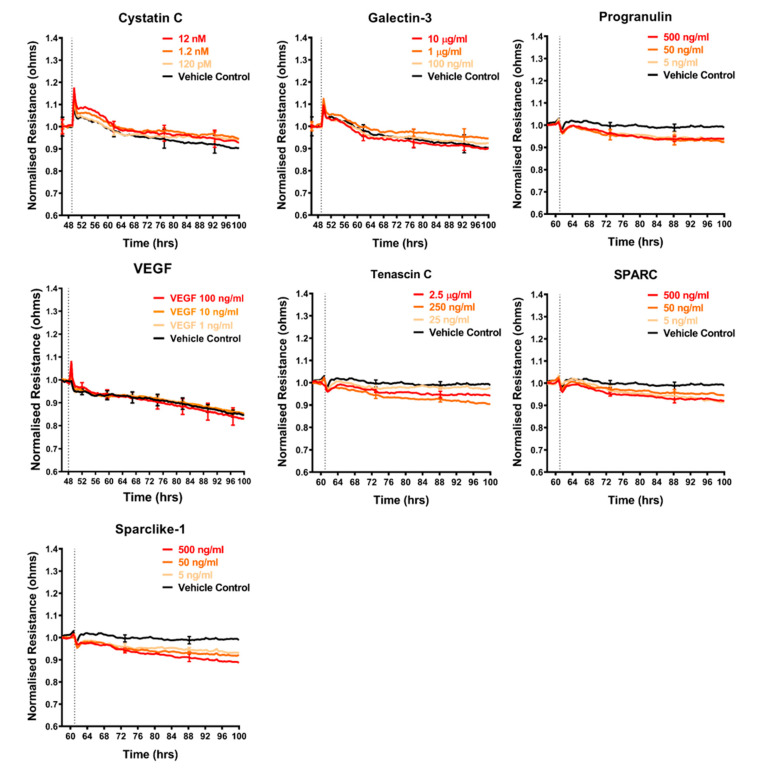
Effect of secreted protein on brain endothelial barrier resistance. Normalised unmodelled resistance (at 4000 Hz) of hCMVECs over time after addition of seven variably expressed secreted proteins. Recombinant proteins were added (dotted line) at top concentrations as recommended by the supplier and literature in a dilution series of 1:10. Data show the mean ± SD (*n* = 3 wells) from one experiment, which is representative of two to three independent experiments.

**Figure 7:**
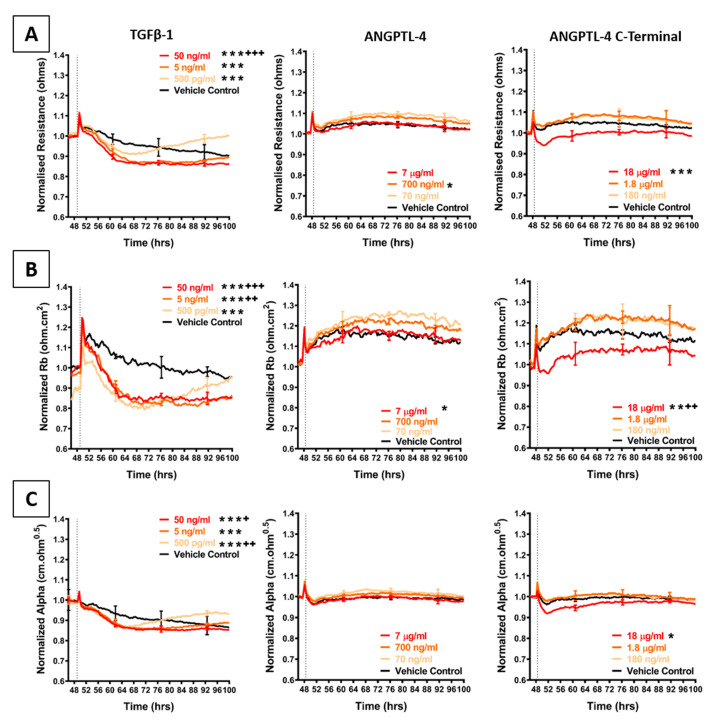
Effect of TGFβ-1 and ANGPTL-4 on brain endothelial barrier resistance. (**A**): shows normalised unmodelled resistance (at 4000 Hz), (**B**): shows modelled Rb (paracellular resistance) and (**C**): shows modelled Alpha (basolateral resistance) of hCMVECs over time after addition of TGFβ-1 and ANGPTL-4 (including the C-terminal fibrinogen like fragment of ANGPTL-4 alone). Recombinant proteins were added (dotted line) at top concentrations as recommended by the supplier and literature in a dilution series of 1:10. Data show the mean ± SD (*n* = 3 wells) from one experiment, which is representative of three to four independent experiments. Values from three independent experiments were compared relative to their media control using two-way ANOVA with Tukey’s range test as detailed in Methods at 60 h (* *p* < 0.05, ** *p* < 0.01, *** *p* < 0.001) and 100 h: (^+^ *p* < 0.05, ^++^ *p* < 0.01, ^+++^ *p* < 0.001).

The shortlist of candidates detailed above was assessed for their direct involvement, to see if any mimicked the effect of the melanoma conditioned media. The recombinant proteins were added individually to the brain endothelial cells and the barrier effects measured by ECIS (Figure 5, Figure 6 and Figure 7). Surprisingly, a number of these recombinant proteins had no detectable effect on the brain endothelial barrier integrity, even at very high concentrations. These included GDF-15, IGFBP-2, MIF, galectin-3, IL-8, and VEGF (Figure 5). Several cytokines may have had a small effect by increasing barrier integrity and these include CXCL-1, DKK-1, and cystatin C (Figure 5 and Figure 6). Angiogenin, progranulin, tenascin C, SPARC, and SPARC-Like-1 marginally decreased barrier resistance (Figure 5 and Figure 6). However, these small responses were not always reproducible across independent experiments.

Figure 7 shows the barrier-mediated responses from TGFβ-1 and the C-terminal fragment of ANGPTL-4. Both of these factors reduced the barrier integrity of the brain endothelial cells consistently across all experiments. For comparison to the C-terminal peptide, full length ANGPLT-4 was also tested, and the full-length version did not disrupt the barrier integrity.

Addition of recombinant TGFβ-1 transiently decreased overall endothelial barrier resistance starting in the first 4–8 h of addition. The decrease was more prominent for Rb, demonstrating that the paracellular barrier was disrupted more than the basolateral barrier. Temporally, the decrease in barrier resistance occurred at the same time for both modelled parameters and was maximal even at the lowest concentration tested (500 pg/mL). Intriguingly, after 24 h of treatment, the barrier resistance increased, suggesting a strengthening of the endothelial barrier. This **increase** occurred in a concentration-dependent manner, wherein addition of TGFβ-1 at the lowest concentration resulted in faster endothelial recovery. Furthermore, the Alpha parameter (basolateral resistance) increased above the vehicle control, suggesting that the basolateral adhesions were stronger post-treatment, compared to control. This was also observed for the paracellular resistance after the 100-h time-point. These results show that the brain endothelial cells are responsive to TGFβ-1 and can recover from TGFβ-1-mediated endothelial disruption. The response to TGFβ-1 is quite distinct to that observed with the melanoma conditioned media.

As TGFβ-1 mediates endothelial destabilisation by initiating the release of ANGPTL-4, this angiopoietin-like protein was also added to the brain endothelial cells. However, the full length ANGPTL-4 did not affect the barrier integrity; whereas, the C-terminal fibrinogen like fragment (cANGPTL-4) had a pronounced effect. cANGPTL-4 was the only assessed secretory molecule, which decreased overall and paracellular (Rb) barrier resistance at the same rate as that seen with the addition of the conditioned media. This effect was only observed at a highest concentration used (18 µg/mL; where the concentrations were determined according to the supplier’s activity recommendations). Over this concentration range, there was minimal change in the basolateral parameter (Alpha). Collectively, these results identify several melanoma secreted cytokines that could be involved in weakening the barrier integrity of brain endothelial cells.

## 3. Materials and Methods

### 3.1. Culture of the Melanoma Cells

Three melanoma cell lines were used in collaboration with Dr Graeme Finlay and Wayne Joseph from the Auckland Cancer Society Research Centre (ACSRC). These were NZM7 (Research Resource ID: CVCL_D843), NZM48 (Research Resource ID: CVCL_S423) and NZM74 (Research Resource ID: CVCL_0D38). The NZM cells were cultured in Falcon 75 cm^2^ tissues culture flasks (T75)(cat# FAL353136) with Minimum Essential Medium α (αMEM, cat#12561072, Gibco, Thermo Fisher Scientific, Waltham, MA, USA) containing 5% FBS (cat# FBSF, Moregate, Bulimba, QLD, Australia), 5 μg/mL insulin, 5 μg/mL transferrin, and 5 ng/mL sodium selenite (ITS, cat#11074547001, Sigma-Aldrich, St. Louis, MO, USA), later referred to as complete αMEM medium [1].

### 3.2. Collection of the Conditioned Media

Conditioned media were collected from three NZM lines (NZM7, NZM48 and NZM74). NZM cells were seeded at a density of 900,000 cells in a T75 flask. The culture medium was collected from the cells from Day 1 (day after cell passage) to Day 7 (~90% confluence) on alternating days. The collected conditioned medium was centrifuged for 10 min at 300× *g* to remove cellular debris and the supernatant was stored at −80 °C.

### 3.3. Culture of the Brain Endothelial Cells

Human cerebral microvascular endothelial cells (hCMVECs) are a cell line, immortalised by SV40 large T antigen, and purchased from Applied Biological Materials Inc. (ABM, cat# T0259, Richmond, BC, Canada) The cells were cultured in Nunc T75 flasks (cat# 156499) and provided with M199 growth medium (cat# 11150-067, Gibco, Thermo Fisher Scientific, Waltham, MA, USA) containing 10% FBS, 1 μg/mL hydrocortisone (cat# H0888, Sigma-Aldrich, St. Louis, MO, USA), 3 ng/mL hFGF (cat# PTAF10018B50, Peprotech, Rocky Hill, NJ, USA), 1 ng/mL hEGF (cat# PTAF10015100, Peprotech, Rocky Hill, NJ, USA), 10 μg/mL heparin (cat# H-3393, Sigma-Aldrich, St. Louis, MO, USA), 2 mM GlutaMAX (cat# 305050-061, Gibco, Thermo Fisher Scientific, Waltham, MA, USA), and 80 μM dibutyryl-cAMP (cat# D0627, Sigma-Aldrich, St. Louis, MO, USA), later referred to as complete M199 medium. For both cell maintenance and experiments, the flasks/plates were coated with 1 μg/cm^2^ collagen I (cat# A1048301, Gibco, Thermo Fisher Scientific, Waltham, MA, USA) dissolved in 0.02 M acetic acid for 1 h and washed three times with sterile MilliQ water, prior to cell plating [1].

### 3.4. ECIS Technology Assessment of Conditioned Media

Electric cell-substrate impedance sensing or ECIS is a real-time and label-free, impedance-based method [69,70]. It allows the assessment of various attributes of cell behaviour such as cell adhesion and barrier strength by measuring the impedance across a confluent cell monolayer. The ECIS theory is detailed thoroughly in [23].

The ECIS Zθ system uses 96-well plates lined with interdigitating fingers of gold-plated electrodes (96W20idf), which were treated with 10 mM cysteine for 15 min to standardise electrode impedance. The wells were coated with 1 µg/cm^2^ of collagen I dissolved in 0.02 M acetic acid according to the cell culture protocol described above. The hCMVECs were seeded at 20,000 cells per well, in 100 µL of complete M199 medium. Cells were allowed to proliferate and form high resistance (measured by increase in ohms) monolayers for approximately 48 h. The ECIS machine was run continuously at multi-frequency, so the ECIS system may record resistance across low and high frequencies and then model the recorded resistance into separate components as developed by Giaever and Keese [71].

On the day of treatment, 100 µL of melanoma conditioned medium was pre-warmed and added to 20,000 hCMVECs per well on the ECIS plate, which had been grown for ~48 h until they had formed a continuous monolayer of high resistance, sustained through their paracellular barrier (Rb). The medium-only control (100 µL of αMEM) was added to the hCMVECs and used to ascertain responses from the conditioned media. All ECIS data were analysed using GraphPad Prism (version 7.03, GraphPad, San Diego, CA, USA) software and plotted as the mean (±SD) of multiple replicates (minimum of three).

### 3.5. Cytokine Measurements Using CBA and Luminex

Expression of secreted proteins in the conditioned media of the NZM cells was quantitatively measured using Cytometric Bead Array (CBA, BD Biosciences, Franklin Lakes, NJ, USA) and Luminex Immunoassay (R&D Systems, Inc., Minneapolis, MN, USA), which are a multiplex bead-based assay that allows testing of different cytokines, chemokines and growth factors of interest, concurrently. The two different assays were used dependent on availability of the purchased protein of interest. On the day of the experiment, a capture bead cocktail, a reporter antibody cocktail and a standard curve were prepared for each secreted protein of interest (detailed in Table A1 in Appendix A, including std. curve range). Samples were prepared according to the manufacturer’s protocol and as detailed in [5,72]. The samples and standards were run on the Accuri C6 flow cytometer and analysed on the FCAP Array v3 software (BD Bioscience, Franklin Lakes, NJ, USA) using the generated standard curve.

### 3.6. NanoString nCounter Analysis

RNA was isolated from the NZM cells using the RNAqueous™ total RNA isolation kit (cat# AM1931, Invitrogen, ThermoFisher, Waltham, MA, USA) according to the manufacturer’s protocol. The RNA quality was assessed using the Agilent TapeStation. RNA samples were assessed for expression of all genes detailed in Table A2, by using the NanoString nCounter^®^ (Seattle, WA, USA). The procedure was conducted by Jason Capedo, of Auckland Clinical Genomics (University of Auckland). The nCounter output of raw mRNA barcode counts was analysed using the nSolver™ Analysis Software v4.0 (NanoString). Herein, the raw mRNA barcode counts were adjusted against background and three housekeeper genes to give the absolute mRNA barcode count for the gene of interest.

### 3.7. Cytokine Screening Using Proteome Profiler Arrays

Proteome Profiler (R&D Systems, Inc., Minneapolis, MN, USA) was used to screen the NZM conditioned media for a large panel of secreted proteins. Two Proteome Profiler arrays were used: Human XL Cytokine Array (cat# ARY022B) and Human XL Oncology (cat# ARY026), according to the manufacturer’s protocol. The Proteome Profiler arrays were imaged using the ChemiDoc™ MP (Bio-rad, Hercules, CA, USA) and ImageLab v6.0.1 software (Bio-rad, Hercules, CA, USA), at an exposure time of 10 s, with signal accumulation. Chemiluminescence spots were analysed to find the first evidence of saturation on the reference spot, and the image collected 120 s after this was used for analysis using ImageJ v1.50i blot analysis.

### 3.8. Assessment of Recombinant Cytokines on Barrier Integrity

Prior to the addition of recombinant proteins (cytokines and growth factors), the hCMVECs had been seeded in 100 μL of complete M199 medium for ~48 h to allow formation of a stable barrier. Recombinant proteins were prepared in complete M199 medium at twice the effective concentration for the experiments. 100 μL of the recombinant proteins were added to the hCMVECs, resulting in a 50% dilution to the final effective concentration. All recombinant proteins are described in Table A3. All ECIS data were analysed using GraphPad Prism software and plotted as the mean (±SD) from multiple replicates (3 wells).

### 3.9. Statistical Analysis

RStudio (version 1.1.414, RStudio, Inc., Boston, MA, USA) and the vascr package developed by James Hucklesby (unpublished) were used to conduct two-way analysis of variance followed by Tukey’s range test. Normality was confirmed using both visual inspection of the data and the Shapiro–Wilk test of normality, whilst homogeneity of variance was confirmed with Levene’s test. All *p* values shown are relative to the vehicle only or media only control at the same time point.

## 4. Discussion

This paper demonstrates that melanoma cells secrete brain endothelial barrier-disrupting factors that may act to weaken the vascular barrier integrity, which we hypothesise would aid the metastatic process and invasion of the brain by cancer cells. Our cytokine and proteome profiling studies have identified several proteins detectable in the conditioned media that directly weaken the barrier integrity. We postulate that some of these factors may be involved and likely work in a regulated coordinated manner to weaken or prepare the brain endothelial barrier for invasion.

In total, we screened the melanoma conditioned media (and cells) for over 150 secreted factors covering classes of key cytokines, chemokines, growth factors, and soluble ligands. This was achieved using a combination of CBA analysis, Luminex assays, Proteome Profiler arrays, and NanoString analysis. Many of the factors were not detected or were present at very low concentrations. Of note was the absence of cytokines such as IL-1β and TNFα, which are well known regulators of barrier permeability. In addition, ANGPTL-4 was only detected in the conditioned media of NZM7 cells so was unlikely to be a candidate. VEGF levels were very low in NZM48 conditioned media, again reducing its likelihood as being a strong contender. In contrast, very high concentrations of IL-8 and osteopontin were detected. These along with the expression of TGFβ-1, as detected by NanoString, collectively made each of these candidates worth assessing further.

The Proteome Profiler data revealed the presence of 95 out of 142 different secreted targets. This was further refined to cytokines/factors that were present at high levels in the conditioned media and expressed by most of the melanoma lines. In total we assessed the specific involvement of 18 different cytokines and soluble factors produced by the melanoma lines. Of the candidates tested, several (including angiogenin, SPARC-like 1, tenascin C and progranulin) induced small negative effects on the brain endothelial barrier integrity. Others including IL-8, osteopontin and MIF, which were detected at high levels, had no effect on the barrier integrity. This was especially surprising for MIF, which mediates endothelial junctional re-arrangement as demonstrated in [62], where the addition of 1 ng/mL rMIF decreased VE-Cadherin expression and increased transmembrane permeability. A more recent study showed that MIF induced endothelial hyper-permeability as measured by impedance sensing on the xCELLigence RTCA system, through a thrombin-mediated process [73]. The experiments were, and typically are, conducted in human skin microvascular endothelial cells (HMEC-1), which are not brain endothelial cells, and this provides the most likely explanation for the discrepancy seen in these results.

The most significant responses were induced by TGFβ-1 and the C-terminal region of ANGPTL-4. The transient effect of TGFβ-1 was intriguing, as it elicited a strengthening of the endothelial barrier over time. O’Carroll et al., 2015, previously showed that inflammatory mediators including TNFα and IL-1β, initially decreased brain endothelial barrier resistance. This decrease was maintained only for a few hours after which the barrier resistance increased substantially in a concentration-dependent manner. The strengthening of the endothelial barrier suggested a pre-existing and developmental protective mechanism of the brain endothelial barrier against inflammatory mediators. The same idea can explain the delayed protective effects observed with TGFβ-1-mediated endothelial barrier disruption and strengthening.

ANGPTL-4 is induced by TGFβ-1 [47] and is implicated in cancer metastasis. Intriguingly, the full length ANGPTL-4 did not reduce barrier integrity. However, the C-terminal fragment (cANGPTL-4) caused an immediate decrease in endothelial barrier integrity, which occurred at the same rate but lesser magnitude as when endothelial cells were exposed to the conditioned media. cANGPTL-4 is produced only from ANGPTL-4. It is cleaved proteolytically from the full length protein by an enzyme family termed proprotein convertase [74], which are typically located intracellularly [75]. Full length ANGPTL-4 was detected in the conditioned media only from NZM7 cells, suggesting that if it was majorly involved, it might also be cleaved intracellularly and secreted as the cANGPTL-4 fragment, which is unlikely to be detected by the Luminex bead-based assay and the Proteome Profilers. We also suspect that this C-terminal fragment may be secreted by melanoma cells locally after addition to the endothelial cells, which is indicative of melanoma cells responding and adapting to the new environment to initiate metastasis, at a substantially fast rate. This research therefore, requires further analysis looking at changes in secretion post co-culture.

The results herein are in accordance with literature that shows that the C-terminal fibrinogen like fragment of ANGPTL-4 can directly bind to VE-Cadherin, Claudin-5 and integrin α5β1 to modulate endothelial junctional integrity [24]. This was also shown to be associated with a decrease in trans-endothelial electrical resistance (TER) of peripheral human endothelial cells, as measured by ECIS after the addition of purified cANGPTL-4 at concentrations ranging over 1–6 µg/mL. Regrettably, no modelled data were shown. In contrast to the findings of Huang et al. [24], in our study, commercial cANGPTL-4 only marginally disrupted the endothelium, albeit immediately, and only at a concentration of 18 µg/mL, which is significantly higher than the concentration of ANGPTL-4 detected in conditioned media collected from Day 7 (<1 ng/mL). Moreover, the conditioned media mediated a decrease in both paracellular and basolateral barrier resistance, demonstrating that if ANGPTL-4 is involved, it is not the only secreted factor present in the conditioned media that disrupted the brain endothelial barrier.

It is possible that the integrated effects of multiple cytokines and factors working in concert could explain the barrier disrupting activity observed with the melanoma conditioned media. For example, the combination of TGFβ-1 and cANGPTL-4, along with some of the other factors may even synergise or mediate a combinatorial response unique to the multiplicity of the cytokine signalling. In addition, the conditioned media may also contain a myriad of other factors such as proteases, lipid mediators and other regulators not covered in our analysis. Therefore, to better understand the involvement of melanoma secreted factors in barrier disruption, further assessment of these soluble factors is required. Cumulatively, our findings disclose that melanoma cells are capable of secreting a broad range of regulatory cytokines, many of which will have key roles in the survival and support of the melanoma cells during metastasis. This study reveals the production of several such cytokines and secreted factors that directly influence the barrier integrity of brain endothelial cells that may have key roles in the brain metastatic process.

## Figures and Tables

**Table 1:** Selected secreted molecules quantified in the melanoma secretome using CBA or Luminex assays.

Secretory Molecule	Common Alias	Function (Relevant to Metastasis or Vascular Modulation)	References
ANGPTL-4	HFARP	TJ disruption	[24]
IFN-γ	-	Lymphocyte extravasation (T_helper_17)	[2,3,25]
IL-1β	-	Influences VE-Cadherin arrangement and increases vascular permeability	[26,27]
IL-4	BSF-1	Vascular hyperpermeability	[28]
IL-6	BSF-2	Elevated in metastatic melanoma	[29,30]
IL-8	CXCL8	Elevated expression closely associated with melanoma invasion and metastasis	[20,31]
IP-10	CXCL10	Lymphocyte extravasation (T-cell)	[4,32]
MCP-1	CCL2	Induces endothelial TJ opening. Vascular permeability	[33,34]
MIP-1α	CCL3	Leukocyte extravasation (Dendritic cell)	[4,35]
MIP-1β	CCL4	Lymphocyte extravasation (T-Cell CD4^+^)	[4,36]
Osteopontin (OPN)	BSP-1	Expression associated with tumour invasion.	[37,38]
SDF-1α	CXCL12	Melanoma matrix invasion	[39]
TGFβ-1	-	Cancer progression, angiogenesis, invasion and metastasis. Osteolytic metastasis in melanoma	[12,40]
VEGF	VPF	Elevated expression closely associated with metastasis. Increases endothelial permeability. Reversible VE-Cadherin endocytosis	[26,41,42,43,44]

**Table 2:** Details of secreted factors detected at relatively high levels across multiple melanoma conditioned media using the Proteome Profilers.

Secretory Molecule	Function (Relevant to Metastasis or Vascular Modulation)	References
Angiogenin	Associated with metastatic potential	[52]
Cystatin C	Implicated in melanoma brain metastasis	[53]
CXCL-1	Tumorigenesis Endothelial activation and leukocyte recruitment	[54,55]
DKK-1	Inhibits melanoma invasiveness Increases platelet mediated endothelial activation	[56,57]
Galectin-3	Contributes to metastasis. Induces endothelial angiogenesis	[58,59]
GDF-15	High expression correlated with reduced overall survival in patients with melanoma	[60]
IGFBP-2	Increased in malignancy	[61]
MIF	Tumour survival Endothelial permeability	[62,63]
Progranulin	VEGF independent angiogenesis. Correlated with melanoma survival	[64,65]
SPARC	Highly implicated in EMT processes and overexpression in melanoma Mediates invasiveness	[66,67]
Tenascin C	Upregulated in melanoma but likely supportive only to cellular adhesion and ECM movement.	[68]

## Appendix A

**Table A1:** Cytokines measured using cytometric bead array (BD Biosciences) and Luminex bead arrays (R&D systems).

Array/Set	Aliases	Assay	Company	Catalogue No.	Standard Curve (pg/mL)
Secreted proteins tested					
Human IL-8	CXCL8	CBA	BD Bioscience	558277	0–5000
Human MCP-1	CCL2	CBA	BD Bioscience	558287	0–5000
Human MIP-1α	CCL3	CBA	BD Bioscience	558325	0–5000
Human VEGF	-	CBA	BD Bioscience	558336	0–5000
Human IL-4	BSF-1	CBA	BD Bioscience	558272	0–5000
Human MIP-1β	CCL4	CBA	BD Bioscience	558288	0–5000
Human IL-6	BSF-2/HGF	CBA	BD Bioscience	558276	0–5000
Human IP-10	CXCL10	CBA	BD Bioscience	558280	0–5000
Human ANGPTL-4	HFARP	Luminex	R&D Systems	LXSAH-4	0–360810
Human Osteopontin	OPN/SPP-1	Luminex	R&D Systems	LXSAH-4	0–636310
Human SDF-1α	CXCl12	Luminex	R&D Systems	LXSAH-4	0–36870
Human IFNγ	IFG	CBA	BD Bioscience	558269	0–5000
Human IL-1β	-	CBA	BD Bioscience	558279	0–5000
TGFβ-1		CBA	BD Bioscience	560429	0–10000
Secreted proteins tested					
Human XL Cytokine Array Kit		Proteome Profiler	R&D Systems	ARY022B	
Human XL Oncology Array Kit		Proteome Profiler	R&D Systems	ARY026	

**Table A2:** Genes assessed by NanoString nCounter analysis.

Protein	Gene	Probe NSID
TGFβ-1	TGFB1	NM_000660.3:1260
VEGF-A	VEGFA	NM_001025366.1:1325
Osteopontin	SPP1	NM_000582.2:760
MCP-1	CCL2	NM_002982.3:123
IL-8	CXCL8	NM_000584.2:25

**Table A3:** Recombinant cytokines and supplier details.

Treatment	Company	Catalogue No.
Angiogenin	R&D	265-AN-050/CF
GDF-15	R&D	8146-GD/CF
Progranulin	R&D	2420-PG-050
SPARC	R&D	941-SP-050
SPARC Like-1	R&D	2728-SL-050
Tenascin C/Cytotactin	R&D	3358-TC-050
IGFBP-2	R&D	674-B2-025
Cystatin C	Biolegend	756202
DKK-1	Biolegend	778602
Galectin-3	Biolegend	599704
MIF	Biolegend	599404
Osteopontin	Biolegend	557102
TGFβ-1	Biolegend	580702
CXCL-1	Biolegend	574402
ANGPTL-4	R&D	RDS4487AN050
cANGPTL-4	R&D	RDS3485AN050
IL-8	PrimeGene	101-08A
VEGF	PrimeGene	105-16Y
